# Genome-Wide Signatures of Selection Detection in Three South China Indigenous Pigs

**DOI:** 10.3390/genes10050346

**Published:** 2019-05-07

**Authors:** Shuqi Diao, Shuwen Huang, Zitao Chen, Jinyan Teng, Yunlong Ma, Xiaolong Yuan, Zanmou Chen, Hao Zhang, Jiaqi Li, Zhe Zhang

**Affiliations:** 1 Guangdong Provincial Key Lab of Agro-Animal Genomics and Molecular Breeding/National Engineering Research Centre for Breeding Swine Industry/College of Animal Science, South China Agricultural University, Guangzhou 510642, China; saradiao@126.com (S.D.); 13422048613@163.com (S.H.); barnettca@outlook.com (Z.C.); kingyan312@live.cn (J.T.); yxl@scau.edu.cn (X.Y.); zmchen@scau.edu.cn (Z.C.); zhanghao@scau.edu.cn (H.Z.); jqli@scau.edu.cn (J.L.); 2 Key Laboratory of Agricultural Animal Genetics, Breeding, and Reproduction of Ministry of Education, College of Animal Sciences and Technology, Huazhong Agricultural University, Wuhan 430070, China; Yunlong.Ma@mail.hzau.edu.cn

**Keywords:** signatures of selection, South China indigenous pigs, SNP, XP-EHH, *F*_ST_

## Abstract

South China indigenous pigs are famous for their superior meat quality and crude feed tolerance. Saba and Baoshan pigs without saddleback were located in the high-altitude area of Yunnan Province, while Tunchang and Ding’an pigs with saddleback were located in the low-altitude area of Hainan Province. Although these pigs are different in appearance, the underlying genetic differences have not been investigated. In this study, based on the single-nucleotide polymorphism (SNP) genotypes of 124 samples, both the cross-population extended haplotype homozygosity (XP-EHH) and the fixation index (*F*_ST_) statistic were used to identify potential signatures of selection in these pig breeds. We found nine potential signatures of selection detected simultaneously by two methods, annotated 22 genes in Hainan pigs, when Baoshan pigs were used as the reference group. In addition, eleven potential signatures of selection detected simultaneously by two methods, annotated 24 genes in Hainan pigs compared with Saba pigs. These candidate genes were most enriched in GO: 0048015~phosphatidylinositol-mediated signaling and ssc00604: Glycosphingolipid biosynthesis—ganglio series. These selection signatures were likely to overlap with quantitative trait loci associated with meat quality traits. Furthermore, one potential selection signature, which was associated with different coat color, was detected in Hainan pigs. These results contribute to a better understanding of the underlying genetic architecture of South China indigenous pigs.

## 1. Introduction

Pigs have been domesticated for 9000 years [1]. During their long history of evolution and breeding, pigs have been selected naturally or artificially for specific traits, such as adaption to high temperature and humidity in South China, coat color, body length, meat quality, and so forth. Many genetic footprints, i.e., signatures of selection, remain in the genome [2,3] and have been of interest for evolutionary biologists and breeders. The study of signatures of selection may provide some information about selection mechanisms and benefit future pig breeding.

The regions of the genome where the signatures of selection can be detected usually show long-range linkage disequilibrium (LD) accompanied by a high population frequency [2,3,4] and these regions can be detected based on genomic data with the population statistical method. Currently, detection approaches for signatures of selection are based on single point frequencies of selected mutations, LD, and population differentiation. Among these, single point frequencies may produce high rates of false positives [5]. Cross-population extended haplotype homozygosity (XP-EHH) [6] is based on the long-range haplotype (LRH) and the integrated haplotype score (iHS) [7], which was applied to identify signatures of selection in cross-populations. The fixation index (*F*_ST_) statistic, which is based on population differentiation, was first defined by Lewontin and Krakauer [8] based on coefficient *F* [9] and was then developed by Weir and Cockerham [10], Akey et al. [11], and Gianola et al. [12].

With the rapid developments in high-throughput sequencing and genotyping, many signatures of selection have recently been detected in the pig genome [13,14,15,16,17]. The study of Rubin et al. [18] showed strong signatures of selection at three loci which harbor *NR6A1*, *PLAG1*, and *LCORL* genes and are associated with the elongation of the back and an increased number of vertebrae in European domestic pigs. Additionally, using the *F*_ST_ statistic, Ai et al. [19] found several genes, including *ADAMTS12*, *SIMI*, and *NOS1,* which are likely associated with adaption to high altitude in Tibetan pigs. Furthermore, some potential signatures of selection related to economic traits, such as disease resistance, pork yield, fertility, tameness, and body length, were found in Berkshire pigs [20]. Compared with Chinese indigenous breeds and commercial pig breeds, the results showed that 81 candidate genes are associated with the development of tissues and organs and the immune response [21].

A few studies [13,21,22,23] have been carried out to detect signatures of selection in Chinese indigenous pigs. Research into breeding goals in Chinese and European domestic pig breeds showed that they all concentrated on genes mostly related to muscle development, the nervous system, and especially to metabolic diseases. The Chinese tend to pay more attention to fat deposits, while Europeans tend to concentrate more on leanness and body length for modern commercial breeds [24]. South China indigenous pig breeds are distributed in the tropical and subtropical areas of South China. South China indigenous pig breeds usually have a white coat with black spots, a black head and haunch, and their body size is usually smaller than other Chinese indigenous pig breeds. Moreover, the backfat of South China pig breeds is thicker [25,26]. Tunchang pigs (a subpopulation of Hainan pigs [25]), Ding’an pigs (a subpopulation of Hainan pigs [25]), Baoshan pigs, and Saba pigs were domesticated in a relatively isolated environment in Hainan Province and Yunnan Province, South China. The long-term geographical and genetic isolation caused differential appearance and potential genetic diversity. Saba and Baoshan pigs, located in a high-altitude area of Yunnan Province (>1500 m above sea level (a.s.l.)), have a black coat without saddleback. Meanwhile, Hainan pigs, located in a low-altitude area, have saddleback. Although the difference in appearance between these two types of pig is easily observed, the underlying genetic differences are yet to be discovered.

The aim of this study was to detect specific signatures of selection associated with the genetic characteristics within the genomes of three breeds of South China indigenous pigs. The XP-EHH test and the *F*_ST_ statistic were used to identify the signatures of selection in South China indigenous pigs using genotype data from the Illumina PorcineSNP60 BeadChip [27] and the GeneSeek Genomic Profiler (GGP) Porcine Chip (https://genomics.neogen.com/en/ggp-porcine). Our findings revealed important candidate functional genes that underwent positive selection in South China indigenous pigs.

## 2. Materials and methods

### 2.1. Ethics Approval

This study was carried out in accordance with the recommendations of the Animal Care Committee of the South China Agricultural University (Guangzhou, People’s Republic of China). The protocol was approved by the Animal Care Committee of the South China Agricultural University (SCAU#2013-10). 

### 2.2. DNA Sample Collection

A total of 124 individuals of three pig breeds (four populations) were collected from four locations in South China. Specifically, 33 Baoshan pigs (BS, 13 males, 20 females, collected from Shidian, Yunnan Province, 16 July 2015), 23 Saba pigs (SB, nine males, 14 females, collected from Chuxiong Yi Autonomous Prefecture, Yunnan Province, 22 July 2015), 34 Ding’an pigs (DA, a subpopulation of the Hainan pig [25], 34 females, collected from Ding’an, Hainan Province, 23 January 2016), and 34 Tunchang pigs (TC, a subpopulation of the Hainan pig [25], 10 males, 24 females, collected from Tunchang, Hainan Province, 10 March 2016).

### 2.3. Single-Nucleotide Polymorphism (SNP) Genotyping and Data Quality Control

Genomic DNA samples from all three breeds of Chinese indigenous pig were extracted from ear tissue using the E.Z.N.A.^®^Tissue DNA Kit (D3396-02, Omega Bio-tek, Norcross, GA, USA). The Illumina PorcineSNP60 BeadChip [27], which contains 61,565 SNPs, was used for the SNP genotyping of Baoshan pigs and Saba pigs, while the GGP Porcine Chip (https://genomics.neogen.com/en/ggp-porcine), which contains 68,516 SNPs, was used for the SNP genotyping of Ding’an pigs and Tunchang pigs. The SNP data of three South China pig breeds are available in the figshare database (https://doi.org/10.6084/m9.figshare.7588235.v1)

The quality control criteria for genotypic data were as follows: (1) Retaining the mutual SNPs between two SNP chips (the alleles of each SNP on two chips were unified according their SNP chip annotation file while merging the genotype from different chips); (2) removing SNP loci with a call rate of less than 0.90 and unknown position or located on sex chromosomes; (3) filtering out individuals with call rates less than 0.90; and (4) removing SNP loci with minor allele frequency (MAF) less than 0.05. PLINK software [28] was used to perform data quality control. Following quality control, fastPHASE [29] was used to infer haplotypes for the haplotype-based method (XP-EHH) with the parameters –KL10, –KU30, and –Ki5.

### 2.4. Principal Component Analysis 

To investigate the pattern of genetic differentiation among breeds, principal component analysis (PCA) was conducted with GCTA software (Version 1.91.1) [30]. Then, the figure of PCA was plotted using R base package with plot function [31]. The SNPs used in this analysis were filtered for pairwise LD (*r^2^* < 0.5) with PLINK software [28] using the command indep-pairwise 50 5 0.5.

### 2.5. Phylogenetic Tree 

In order to better understand the relationship between the three breeds investigated in this study, a phylogenetic tree based on the pairwise identical by state (IBS) was constructed. The average proportion of alleles shared among all individuals (denoted as Dst) was calculated as follows: Dst=(IBS2+0.5×IBS1)/N, where IBS1 and IBS2 are the number of loci which share either one or two alleles IBS of two individuals, respectively, and N is the total number of SNPs. Then, 1- Dst is the genetic distance between all pairwise combinations of individuals, as in Ai et al. [19]. The Dst was calculated by PLINK software [28]. A neighbor-joining (N-J) tree [32] based on genetic distance was constructed by MEGA software (Version 7.0.14) [33].

### 2.6. Identification of Signatures of Selection

Both the XP-EHH and the *F*_ST_ were used for the detection of signatures of selection in this study. The XP-EHH was needed to define test groups and reference groups. In this study, XP-EHH [34] was used to calculate the XP-EHH scores. A chromosome segment of 1 Mb was directly converted as 1 centiMorgan (cM) in Ma et al. [23]. The Genepop R package [35] was used to calculate *F*_ST_ statistics. The *F*_ST_ statistic indicated the population differentiation; however, it was unable to indicate which population experienced selection.

In previous research, XP-EHH scores were reported to approximately follow a normal distribution [6]. In this study, the unstandardized XP-EHH scores were transformed into a normal distribution. Then, the *p*-values of standardized XP-EHH scores which were lower than 0.01 were treated as significant SNPs detected by XP-EHH, as in Li et al. [13]. The distribution of *F*_ST_ approximately followed a normal distribution after the normalization of the square root of *F*_ST_, as in Gianola et al. [12]. In this study, the *p*-values of standardized *F*_ST_ below 0.01 were treated as significant SNPs. *F*_ST_ may produce high rates of false positives compared with XP-EHH, as suggested by Ma et al. [36]. Therefore, the significant SNPs detected either both methods or at least one method, which were treated as potential signatures of selection in this study. In addition, this study focused on the significant SNPs detected simultaneously by two methods.

### 2.7. Genome Annotation and Quantitative Trait Loci (QTL) Overlapping with Potential Signatures of Selection

The potential selection regions were defined by extending 200 kb both upstream and downstream of the potential signatures of selection in Liu et al. [37]. Genome annotation was based on the *Sus scrofa* 10.2 (https://www.animalgenome.org/blast/). Genes harbored in these potential selection regions were treated as candidate genes and RNAs and unconfirmed genes were filtered out. Additionally, the Animal Quantitative Trait Loci (QTL) Database [38] was used to annotate potential traits related to the potential selection regions based on QTL physical position intervals downloaded from the Animal QTL database [38].

### 2.8. Gene Ontology (GO) Terms and Kyoto Encyclopedia of Genes and Genomes (KEGG) Pathway Enrichment Analysis

To further explore the function of these candidate genes, Kyoto Encyclopedia of Genes and Genomes (KEGG) pathway [39] and Gene Ontology (GO) [40] were used for enrichment analyses through the Database for Annotation, Visualization, and Integrated Discovery (DAVID) Version 6.8 (https://david.ncifcrf.gov/) [41,42]. The GO terms and KEGG pathways with *p*-values > 0.05 were filtered out.

## 3. Results

### 3.1. Genotypes and Population Structure

A total of 34,815 SNPs located on autosomes were common in the two SNP chips. In quality control, 1833 and 9444 SNPs were filtered out for the SNP call rate and MAF, respectively. The average individual call rate was 0.9781 and no individual was removed. After quality control, 124 individuals and 23,538 SNPs were retained for further study.

**Figure 1:**
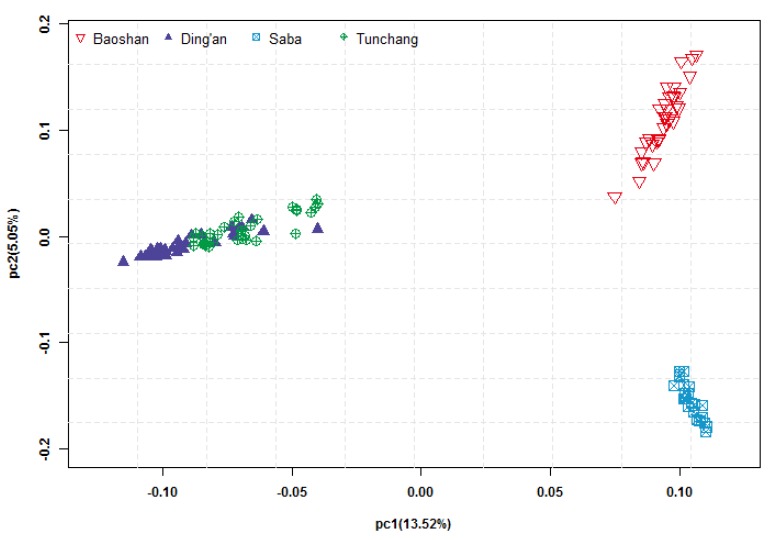
Principal component analysis of 33 Baoshan pigs (red triangles), 23 Saba pigs (blue squares), 34 Ding’an pigs (purple triangles), and 34 Tunchang pigs (green targets). The principal component analysis was conducted by GCTA software (Version 1.91.1) [30], plotted with R base package plot function [31].

**Figure 2:**
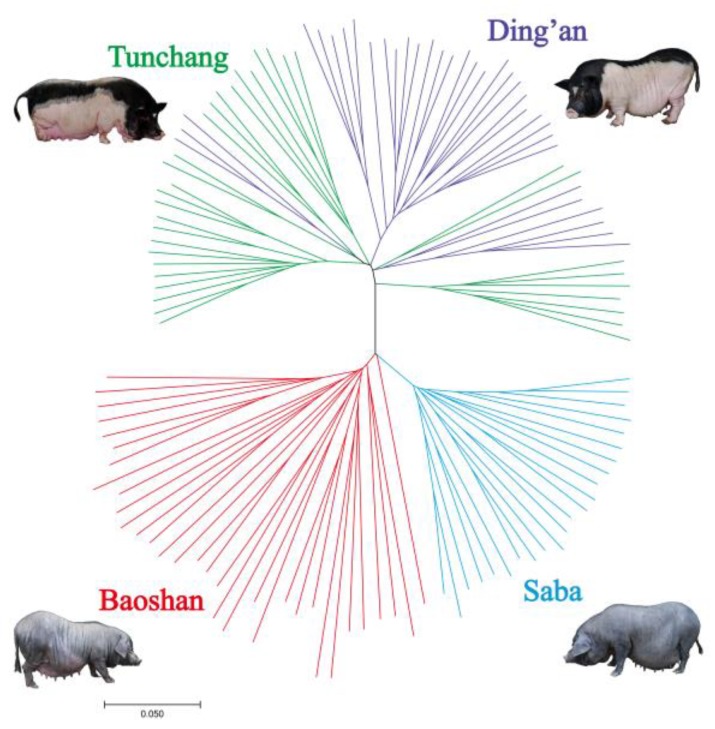
Phylogenetic tree based on four pig populations. The polygenetic tree was constructed based on data collected from 33 Baoshan pigs, 23 Saba pigs, 34 Ding’an pigs, and 34 Tunchang pigs. Different colors represent different pig populations. The phylogenetic tree was constructed by MEGA software (Version 7.0.14) [33].

A subset of 23,538 SNPs (18,994 LD-pruned SNPs) were retained to conduct PCA. PCA1 and PCA2 explained 13.52% and 5.05% of the total variation, respectively (Figure 1). Individuals of two Hainan pig subpopulations were clustered together, as also shown by the N-J tree (Figure 2). Baoshan and Saba appeared as two separate groups. The average genetic distance between Baoshan pigs (0.2775 ± 0.0042) was the highest among the four pig populations (Saba: 0.2213 ± 0.0056; Ding’an: 0.2209 ± 0.0067; Tunchang: 0.2345 ± 0.0062). More details are shown in Table S1.

### 3.2. Identification of Signatures of Selection 

According to the result of the PCA (Figure 1) and the N-J tree (Figure 2), the two subpopulations of Hainan pigs (Ding’an pigs and Tunchang pigs) were treated as test groups, while the Baoshan and Saba populations were used as reference groups, respectively.

**Figure 3:**
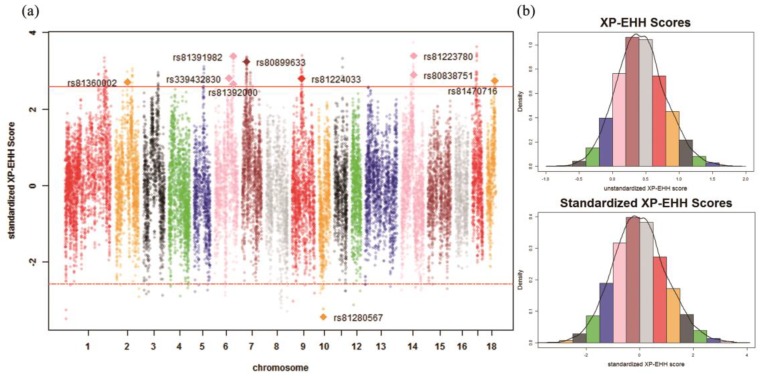
Cross-population extended haplotype homozygosity (XP-EHH) scores across all autosomes in the Ding’an and Tunchang/Baoshan groups. (**a**) Genome-wide distribution of signatures of selection detected by XP-EHH across all autosomes in the Ding’an and Tunchang/Baoshan groups. The SNPs shown as diamonds are the significant SNPs detected simultaneously by two methods. The red lines show the threshold *p*-value (0.01). (**b**) The distribution of unstandardized XP-EHH scores and standardized XP-EHH scores across all autosomes in the Ding’an and Tunchang/Baoshan groups.

**Figure 4:**
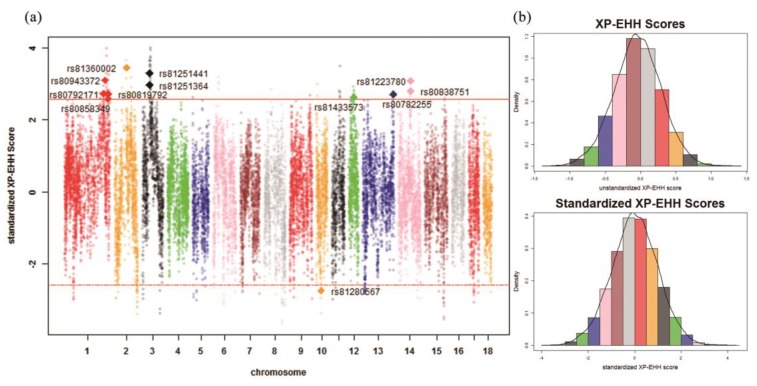
XP-EHH scores across all autosomes in the Ding’an and Tunchang/Saba groups. (**a**) Genome-wide distribution of signatures of selection detected by XP-EHH across all autosomes in the Ding’an and Tunchang/Saba groups. The SNPs shown as diamonds are the significant SNPs detected simultaneously by two methods. The red lines show the threshold *p*-value (0.01). (**b**) The distribution of unstandardized XP-EHH scores and standardized XP-EHH scores across all autosomes in the Ding’an and Tunchang/Saba groups.

**Figure 5:**
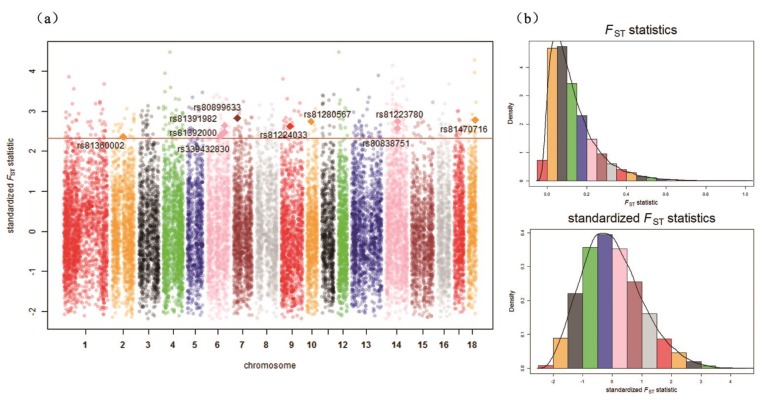
*F*_ST_ statistics across all autosomes in the Ding’an and Tunchang/Baoshan groups. (**a**) Genome-wide distribution of signatures of selection detected by *F*_ST_ statistics across all autosomes in the Ding’an and Tunchang/Baoshan groups. The SNPs shown as diamonds are the significant SNPs detected simultaneously by two methods. The red line shows the threshold *p*-value (0.01). (**b**) The distribution of unstandardized *F*_ST_ statistics and standardized *F*_ST_ statistics across all autosomes in the Ding’an and Tunchang/Baoshan groups.

**Figure 6:**
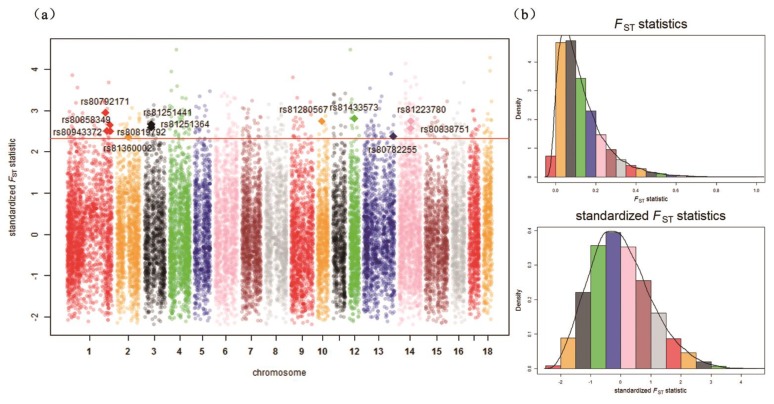
*F*_ST_ statistics across all autosomes in the Ding’an and Tunchang/Saba groups. (**a**) Genome-wide distribution of signatures of selection detected by *F*_ST_ statistics across all autosomes in the Ding’an and Tunchang/Saba groups. The SNPs shown as diamonds are the significant SNPs detected simultaneously by two methods. The red line shows the threshold *p*-value (0.01). (**b**) The distribution of unstandardized *F*_ST_ statistics and standardized *F*_ST_ statistics across all autosomes in the Ding’an and Tunchang/Saba groups.

The distribution of unstandardized XP-EHH scores and standardized XP-EHH scores are shown in Figure 3b and Figure 4b. The distribution of *F*_ST_ statistics and standardized *F*_ST_ statistics are shown in Figure 5b and Figure 6b. In the Ding’an and Tunchang/Baoshan groups, 174 SNPs and 71 SNPs were treated as significant through XP-EHH in Hainan pigs and Baoshan pigs (Table S2). Meanwhile, 445 SNPs were treated as significant using *F*_ST_ (Table S2). Similarly, in the Ding’an and Tunchang/Saba groups, 110 SNPs and 125 SNPs were treated as significant through XP-EHH in Hainan pigs and Baoshan pigs (Table S3). Meanwhile, 445 SNPs were treated as significant using *F*_ST_ (Table S3). 

On one hand, nine and eleven potential signatures of selection were detected simultaneously by two methods in Hainan pigs using Baoshan and Saba pigs as the reference groups, respectively. Moreover, compared to the reference group, three significant SNPs were detected simultaneously by two methods in both of the two comparisons (Ding’an and Tunchang/Baoshan, Ding’an and Tunchang/Saba), which were located on *Sus scrofa* chromosome 2 (SSC2) (rs81360002) and SSC14 (rs81223780 and rs80838751), respectively. Furthermore, rs81280567 was the only significant SNP detected simultaneously by two methods in both Baoshan and Saba pigs (Table 1 and Table 2). On the other hand, a total of 680 potential signatures of selection were detected by at least one method in Hainan pigs and Baoshan pigs (Table S2). In addition, the mean LD degree between pairs of significant SNPs detected by at least one method was 0.1626; furthermore, a total of 35 pairs of significant SNPs detected by at least one method (a majority of SNPs located in SSC14) were in high LD ($r^{2}>\;0.8$) (Table S2 and Figure S1). A total of 668 potential signatures of selection were detected by at least one method in Hainan pigs and Saba pigs (Table S3). In addition, the mean LD degree between pairs of significant SNPs detected by at least one method was 0.1665; furthermore, a total of 31 pairs of significant SNPs detected by at least one method (a majority of SNPs located in SSC14) were in high LD ($r^{2}>\;0.8$) (Table S3 and Figure S2).

### 3.3. Genome Annotation and QTL Overlapping with Potential Signatures of Selection

Within the potential selection regions detected simultaneously by two methods in the Ding’an and Tunchang groups, 22 and 24 candidate genes were annotated in the National Coalition Building Institute database, respectively. QTL overlapping with the potential selection regions detected simultaneously by two methods was associated with backfat at last rib, average backfat thickness, drip loss, and so on, as shown in Table 1 and Table 2 and Table S4 and Table S6. More details for each QTL trait are described in the animal QTL database (https://www.animalgenome.org/QTLdb/). Two candidate genes were annotated in the two reference groups (Baoshan and Saba). The one potential selection region detected simultaneously by two methods overlapped with QTLs related to average daily gain, dressing percentage, percentage type I fibers, and so on (see Table 1 and Table 2 and Table S5 and Table S7).

In addition, a total of 1349 candidate genes annotated in 680 significant SNPs detected by at least one method in Hainan pigs and Baoshan pigs and the QTLs overlapping with these significant SNPs were associated with meat quality, average backfat thickness, and so on (Table S2), while 1267 candidate genes annotated in 668 significant SNPs detected by at least one method in Hainan pigs and Saba pigs totally and the QTLs overlapping with these significant SNPs were associated with meat quality, average daily gain, and so on (Table S3).

### 3.4. GO Terms and KEGG Pathway Enrichment Analysis

Within the potential selection regions detected simultaneously by two methods in the Ding’an and Tunchang groups, 41 candidate genes were annotated in total (Table 1 and Table 2). One GO term and one KEGG pathway were enriched and targeted, both of which involved two candidate genes (Table 3). The enriched KEGG pathway was ssc00604 (glycosphingolipid biosynthesis—ganglio series) and the enriched GO term was GO: 0048015 (phosphatidylinositol-mediated signaling). However, no GO term or KEGG pathway was enriched in the reference group (Baoshan, Saba). 

The 1349 candidate genes annotated in 680 significant SNPs detected by at least one method in Hainan pigs and Baoshan pigs involved 22 GO terms and 12 KEGG pathways (Table S2) and 1267 candidate genes annotated in 668 significant SNPs detected by at least one method in Hainan pigs and Saba pigs involved 13 GO terms and 17 KEGG pathways (Table S3).

## 4. Discussion

In this study, the potential signatures of selection in South China indigenous pig populations were identified using two approaches. Nine and eleven potential signatures of selection were detected simultaneously by two methods in Hainan pigs with Baoshan and Saba pigs as reference groups, respectively. Moreover, 22 and 24 candidate genes were found to be enriched in Hainan pigs with Baoshan and Saba pigs as reference groups, respectively. These selection regions were overlapping with QTLs associated with meat quality, disease resistance, and growth. In Baoshan and Saba pigs, only one potential signature of selection was detected simultaneously by two methods was identified, which overlapped with growth and meat quality traits. These results together suggest the potential utility of the findings from the present study.

In general, the signatures of selection revealed by the methods based on population differentiation were associated with phenotypic changes in morphology and behavior. Interestingly, there was a significant difference in coat color between Hainan pigs and the reference populations. Furthermore, a potential signature of selection on SSC14 (rs81223780) was detected in Hainan pigs with either Baoshan or Saba pigs as the reference population. This region was reported by Wilkinson et al. [43] when black and partially black coat breeds (Large Black, Berkshire, Hampshire, British Saddleback) were compared against red coat breeds (Duroc). The results from the present study, together with those of Wilkinson et al. [43], suggest that a promising functional candidate region for pig coat color has been identified and that this region could be of research interest in the future. Additionally, one candidate gene associated with coat color (melanocortin 5 receptor, *MC5R*) was detected in this study. The *MC5R* gene, a member of the melanocortin receptor gene family, has been reported to create a ligand-dependent signal modulation with *MC1R*, which may participate in physiological color change in flounder [44]. Moreover, another study reported that the *MC5R* gene polymorphism (A303G) may affect the feed intake, feed conversion, and other physicochemical characteristics in Large White x Landrace crossbred pigs [45]. 

The phenotypes of each individual were not included in most of the signatures of selection detection analysis, hence the functional explanation of significant signals was usually less conclusive. Although a new methodology for the detection of signature of selection for specific complex traits was recently proposed by Beissinger et al. [46], the phenotypic values were not always available in such research. The reported QTLs could serve as a reference or potential clue to understand the identified signatures of selection. In this study, the Animal QTL database [38] and enrichment analysis were used to enhance our understanding of the detected signature of selection. The QTLs overlapping with potential selection regions were mainly related to traits of meat quality, disease resistance, and growth. It is known that the meat quality of most Chinese indigenous pigs is superior, especially for Ding’an and Tunchang pigs [25,26]. Traditionally, the priorities of pig domestication in China were fat deposit and reproduction, which was confirmed by Wang et al. [24]. From this perspective, the signatures of selection detected in this study would be related to those traits annotated from the above analysis. However, we should be sufficiently cautious to conduct further specific functional research based solely on the findings from signature detection. Pavlidis et al. [47] reported that annotation term enrichment is known to not perform well when applied to selective sweeps.

Although some interesting findings were reported here, the limitations of the present study should not be neglected. These include: (1) The low density of markers. The average distance between adjacent SNPs is 100 kb and the average LD degree between pairs of SNPs with a distance within 200 kb of each population is 0.201, 0.168, 0.151, and 0.188 in Ding’an, Tunchang, Baoshan, and Saba pigs, respectively. This indicates that the SNPs were not dense enough in the present study, although similar SNP chips were used in other studies [23]; (2) the effectiveness of the two detection methods used in this study. The *F*_ST_ method may bring a higher false positive rate compared with XP-EHH, as suggested by Ma et al. [23]. Furthermore, the *F*_ST_ is suitable for the detection of genome regions that are differentially fixed in different breeds, while XP-EHH is used in detecting variants which are still segregating in populations and are a subject of ongoing selection. In addition, focusing on SNPs detected by both methods, some potential signatures of selection among breeds might be neglected and the combination of significant SNPs detected by one method might cause false positive results. To provide comprehensive and balanced results, the significant SNPs detected by either two methods or at least one method were provided and analyzed simultaneously in this study; (3) the small size of the effective population of the three breeds might affect the *F*_ST_ statistic; and (4) the contrast between Yunnan and Hainan pigs was insufficient. Although geographic isolation exists, the direction of Chinese pig domestication was similar in different regions. These limitations together might impact the observations of this study and should be overcome in further investigations.

In conclusion, some potential signatures of selection that might be functionally associated with meat quality, disease resistance, and growth were detected in Hainan pig genomes. Moreover, potential signatures of selection and two candidate genes were detected in Saba and Baoshan pig populations. This study may provide knowledge for the genetic foundation of adaptive evolution in three breeds of South China indigenous pigs.

## Figures and Tables

**Table 1:** Summary of significant single-nucleotide polymorphisms (SNPs) detected simultaneously by two methods in the Ding’an and Tunchang/Baoshan pig groups.

Chr. ^1^	ID	Detected in	iHH ^2^ in Test ^3^	iHH in Ref ^4^	Standardized XP-EHH ^5^ Score	*F*_ST_	Genes	QTL ^6^ (Counts)
2	rs81360002	Test	3.2266	0.7763	2.7056	0.4685	*BTF3, ANKRA2, UTP15, ARHGEF28*	*Actinobacillus pleuropneumoniae* susceptibility (7)
6	rs339432830	Test	3.9553	0.9181	2.8027	0.4726	*MC5R, RNMT, FAM210A, LDLRAD4, CEP192*	Backfat at last rib (11)
6	rs81391982	Test	1.0048	0.1885	3.3794	0.5273	*PIK3C3*	Backfat at last rib (9)
6	rs81392000	Test	0.9475	0.2324	2.6532	0.4930	*–*	Backfat at last rib (9)
7	rs80899633	Test	1.3000	0.2568	3.2394	0.5677	*GRM4, HMGA1, NUDT3, RPS10, PACSIN1, SPDEF*	Average backfat thickness (20)
9	rs81224033	Test	1.7863	0.4160	2.7937	0.5233	*PLEKHA6, PPP1R15B, PIK3C2B, MDM4, LRRN2*	Shoulder weight (3)
14	rs81223780	Test	2.4569	0.4583	3.3948	0.5498	*NRG3*	Fat androstenone level (4)
14	rs80838751	Test	2.6767	0.5997	2.8987	0.5129	*NRG3*	Fat androstenone level (4)
18	rs81470716	Test	0.7901	0.1880	2.7350	0.5564	*–*	*Actinobacillus pleuropneumoniae* susceptibility (3)
10	rs81280567	Ref	0.1573	0.3664	–3.4439	0.5492	*FRMD3, RASEF*	Average daily gain (4)

^1^ Chromosome; ^2^ the integrated haplotype score; ^3^ test group (Ding’an and Tunchang pigs); ^4^ reference group (Baoshan pigs); ^5^ cross-population extended haplotype homozygosity (XP-EHH); ^6^ QTL: Quantitative trait loci—the traits with the highest QTL count are shown here and all QTLs can be seen in Table S4 and Table S5.

**Table 2:** Summary of significant SNPs detected simultaneously by two methods in the Ding’an and Tunchang/Saba pig groups.

Chr. ^1^	ID	Detected in	iHH ^2^ in Test ^3^	iHH in Ref ^4^	Standardized XP-EHH ^5^ Score	*F*_ST_	Genes	QTL ^6^ (Counts)
1	rs80792171	Test	1.3676	0.5713	2.7257	0.5956	*LRSAM1, FAM129B, STXBP1, CFAP157, PTRH1, TTC16, TOR2A, SH2D3C, CDK9, FPGS, ENG, AK1, ST6GALNAC6, ST6GALNAC4*	Drip loss (15)
1	rs80943372	Test	4.5343	1.6659	3.1073	0.4985	*–*	Drip loss (16)
1	rs80858349	Test	2.2043	0.9644	2.5884	0.5304	*–*	Drip loss (16)
1	rs80819792	Test	0.9818	0.4111	2.7187	0.4975	*–*	Drip loss (16)
2	rs81360002	Test	3.2297	1.0560	3.4540	0.4685	*BTF3, ANKRA2, UTP15, ARHGEF28*	*Actinobacillus pleuropneumoniae* susceptibility (7)
3	rs81251364	Test	1.8817	0.7229	2.9747	0.5172	*UXS1, C3H2orf40, NCK2*	Average daily gain (6)
3	rs81251441	Test	1.8762	0.6455	3.3025	0.5333	*UXS1, C3H2orf40, NCK2*	Average daily gain (6)
12	rs81433573	Test	0.9817	0.4227	2.6354	0.5630	*ANKFN1, NOG*	Muscle moisture percentage (4)
13	rs80782255	Test	1.0648	0.4471	2.7108	0.4731	*–*	Body weight (5 weeks) (1)
14	rs81223780	Test	2.5220	0.9360	3.0773	0.5498	*NRG3*	Fat androstenone level (4)
14	rs80838751	Test	2.7541	1.1181	2.8106	0.5129	*NRG3*	Fat androstenone level (4)
**1**0	rs81280567	Ref	0.1720	0.4522	–2.7414	0.5492	*FRMD3, RASEF*	Average daily gain (4)

^1^ Chromosome; ^2^ the integrated haplotype score; ^3^ test group (Dign’an and Tunchang pigs); ^4^ reference group (Saba pigs); ^5^ cross-population extended haplotype homozygosity (XP-EHH); ^6^ the traits with the highest QTL count are shown here and all QTLs can be seen in Table S6 and Table S7.

**Table 3:** Gene Ontology (GO) terms and Kyoto Encyclopedia of Genes and Genomes (KEGG) pathways enriched with candidate genes in Hainan pig populations.

GO Terms and KEGG Pathways	Count	Genes	*p*-Value	Test/Ref ^1^
GO: 0048015~phosphatidylinositol-mediated signaling	2	*PIK3C2B, PIK3C3*	0.0215	Ding’an and Tunchang/Baoshan
ssc00604: Glycosphingolipid biosynthesis—ganglio series	2	*ST6GALNAC6, ST6GALNAC4*	0.0198	Ding’an and Tunchang/Saba

^1^ test/ reference group.

## Supplementary Materials

The following are available online at https://www.mdpi.com/2073-4425/10/5/346/s1, Figure S1: LD plot of significant SNPs in Hainan pigs and Baoshan pigs, Figure S2: LD plot of significant SNPs in Hainan pigs and Saba pigs, Table S1: Genetic distance within populations, Table S2: Significant SNPs detected by single method in Hainan pigs and Baoshan pigs, Table S3: Significant SNPs detected by single method in Hainan pigs and Saba pigs, Table S4: Summary of potential selection regions in Hainan pigs (Ding’an and Tunchang/Baoshan group), Table S5: Summary of potential selection regions in Baoshan pigs (Ding’an and Tunchang/Baoshan group); Table S6: Summary of potential selection regions in Hainan pigs (Ding’an and Tunchang/Saba group), Table S7: Summary of potential selection regions in Saba pigs (Ding’an and Tunchang/Saba group).
