# Supplementary material for: Genome-Wide Signatures of Selection Detection in Three South China Indigenous Pigs
Source: Genes (Basel). 2019 May 7;10(5):346. doi: 10.3390/genes10050346 (PMC6563113; doi:10.3390/genes10050346)
Supplement: Supplementary file 1 [file genes-10-00346-s001.zip › Supplementary-Tables4-7.docx]

**Table S4.** Summary of potential selection regions in Hainan pigs (Ding’an and Tunchang/Baoshan group).

| **Chr^1^** | **SNP** | **ID** | **Position (Mb)** | **XP-EHH**  **score** | **Genes** | **QTL**  **(counts)** |
| --- | --- | --- | --- | --- | --- | --- |
| 2 | H3GA0006932 | rs81360002 | 82.66-83.06 | 1.4247 | *BTF3, ANKRA2, UTP15, ARHGEF28* | Actinobacillus pleuropneumoniae susceptibility (7), C3c concentration (4), Carcass weight (hot) (3), Cooking loss (3), Ham weight (3), Loin muscle area (3), Shear force (3), Shoulder subcutaneous fat thickness (3), Abdominal fat weight (2), average daily gain (2), backfat at last rib (2), backfat at mid-back (2), Drip loss (2), Estimated carcass lean content (2), Fat to meat ratio (2), Firmness (2), Off-Flavor Score (2), PH for Longissmus dorsi (2), tenderness score (2), Water holding capacity (2), androstenone laboratory (1), Average backfat thickness (1), Backfat at first rib (1), Backfat weight (1), Belly meat content (1), Body weight (16 days) (1), Body weight (end of test) (1), Carcass length (1), Carcass temperature (45 minutes post-mortem) (1), Carcass weight (cold) (1), Cholesterol level (1), CIE-b* (1), CIE-L* (1), Creatine kinase level (1), Cutlet weight (1), Diameter of muscle fibers (1), Diameter of type IIa muscle fibers (1), Diameter of type IIb muscle fibers (1), Fat-cuts percentage (1), Ham fat thickness (1), Intramuscular fat content (1), Loin muscle depth (1), Lumbar vertebra number (1), Meat color-L (1), Melanoma susceptibility (1), Muscle moisture percentage (1), Mycoplasma hyopneumoniae antibody titer (1), pH 24 hr post-mortem (loin) (1), pH 24 hr post mortem (ham) (1), pH for Semimembranosus (1), Platelet count (1), Red cell distribution width (1), Salmonella count in liver and spleen (1), Salmonella count in liver (1), Shear firmness (1), Shoulder external fat weight (1), Shoulder weight (1), Tetanus antibody titer (1) |
| 6 | DIAS0000866 | rs339432830 | 96.32-96.72 | 1.4605 | *MC5R, RNMT, FAM210A, LDLRAD4,CEP192* | backfat at last rib (11), Backfat at tenth rib (8), Loin muscle area (7), Fat area percentage in carcass (6), Intramuscular fat content (6), average daily gain (4), Carcass length (4), CIE-a* (4), PH for Longissmus dorsi (4), Carcass weight (hot) (3), muscle protein percentage (3), pH for Semimembranosus (3), Alkaline phosphatase activity (2), Average backfat thickness (2), Conductivity 24 hours postmortem (loin) (2), Conductivity 45 minutes post-mortem (2), Daily feed intake (2), Empty body lipid content (2), Fat weight (total) (2), Ham weight (2), Muscle moisture percentage (2), Platelet count (2), Teat number (2), Abdominal fat weight (1), Age at puberty (1), Backfat between 3rd and 4th last ribs (1), Backfat linear at last rib (1), Backfat linear at tenth rib (1), Belly weight (1), body weight (10 weeks) (1), body weight (13 weeks) (1), body weight (17 weeks) (1), Body weight (20 weeks) (1), Body weight (3 weeks) (1), Body weight (birth) (1), Body weight (end of test) (1), Calcium level (1), Carcass temperature (24 hr post-mortem) (1), Cholesterol level in meat (1), Cholesterol level (1), CSFV antibody level (1), Ear erectness (1), External fat on loin (1), Flavor score (1), Ham fat thickness (1), hematocrit (1), Hemoglobin (1), Hemolytic complement activity (classical pathway) (1), Interleukin 2 level (1), Lactate dehydrogenase level (1), Lean meat percentage (1), Leptin level (1), Litter size (1), Loin fat percentage (1), Loin weight (1), Marbling (1), Mean corpuscular hemoglobin content (1), mummified pigs (1), Nonfunctional nipples (1), Oleic acid content (1), post-stress mitogen induced IL-2 activity (1), Red blood cell count (1), Salmonella count in spleen (1), Shear force (1), Shoulder meat weight (1), Shoulder weight (1), skatole laboratory (1), skatole sensory panel (1), Smell intensity (1), Spareribs weight (1), subjective boar flavor in lean (1), tenderness score (1), Time spent feeding (1), White blood cell counts (1) |
| 6 | ALGA0036875 | rs81391982 | 125.8-126.2 | 1.6734 | *PIK3C3* | backfat at last rib (9), Backfat at tenth rib (8), Intramuscular fat content (8), Loin muscle area (6), CIE-a* (4), Fat area percentage in carcass (4), Marbling (3), muscle protein percentage (3), Teat number (3), Alkaline phosphatase activity (2), Average backfat thickness (2), Carcass weight (hot) (2), Daily feed intake (2), Empty body lipid content (2), Fat-cuts percentage (2), Fat weight (total) (2), Ham weight (2), Platelet count (2), Age at puberty (1), average daily gain (1), Backfat between 3rd and 4th last ribs (1), Backfat linear at last rib (1), Backfat linear at tenth rib (1), Belly weight (1), body weight (10 weeks) (1), Body weight (20 weeks) (1), Body weight (3 weeks) (1), Body weight (birth) (1), Body weight (end of test) (1), Calcium level (1), Carcass length (1), Carcass temperature (24 hr post-mortem) (1), Cholesterol level in meat (1), Ear erectness (1), External fat on loin (1), Flavor score (1), Ham fat thickness (1), hematocrit (1), Hemoglobin (1), Leptin level (1), Litter size (1), Loin fat percentage (1), Loin weight (1), Muscle moisture percentage (1), Oleic acid content (1), post-stress mitogen induced IL-2 activity (1), Red blood cell count (1), Salmonella count in spleen (1), Shear force (1), Shoulder meat weight (1), Shoulder weight (1), skatole laboratory (1), skatole sensory panel (1), Smell intensity (1), Spareribs weight (1), subjective boar flavor in lean (1), Time spent feeding (1) |
| 6 | ALGA0036880 | rs81392000 | 126.18-126.58 | 1.4053 | *--* | backfat at last rib (9), Backfat at tenth rib (8), Intramuscular fat content (8), Loin muscle area (6), CIE-a* (4), Fat area percentage in carcass (4), Marbling (3), muscle protein percentage (3), Teat number (3), Alkaline phosphatase activity (2), Average backfat thickness (2), Carcass weight (hot) (2), Daily feed intake (2), Empty body lipid content (2), Fat-cuts percentage (2), Fat weight (total) (2), Ham weight (2), Platelet count (2), Age at puberty (1), average daily gain (1), Backfat between 3rd and 4th last ribs (1), Backfat linear at last rib (1), Backfat linear at tenth rib (1), Belly weight (1), body weight (10 weeks) (1), Body weight (20 weeks) (1), Body weight (3 weeks) (1), Body weight (birth) (1), Body weight (end of test) (1), Calcium level (1), Carcass length (1), Carcass temperature (24 hr post-mortem) (1), Cholesterol level in meat (1), Ear erectness (1), External fat on loin (1), Flavor score (1), Ham fat thickness (1), hematocrit (1), Hemoglobin (1), Leptin level (1), Litter size (1), Loin fat percentage (1), Loin weight (1), mummified pigs (1), Muscle moisture percentage (1), Oleic acid content (1), post-stress mitogen induced IL-2 activity (1), Red blood cell count (1), Salmonella count in spleen (1), Shear force (1), Shoulder meat weight (1), Shoulder weight (1), skatole laboratory (1), skatole sensory panel (1), Smell intensity (1), Spareribs weight (1), subjective boar flavor in lean (1), Time spent feeding (1) |
| 7 | MARC0076544 | rs80899633 | 30.13-30.53 | 1.6217 | *GRM4, HMGA1, NUDT3, RPS10, PACSIN1, SPDEF* | Average backfat thickness (20), average daily gain (15), backfat at last rib (7), Conductivity 45 minutes post-mortem (6), backfat at mid-back (5), Carcass length (5), Fat androstenone level (5), NADP-malate dehydrogenase activity (5), Backfat at last lumbar (4), CIE-a* (4), Adipocyte diameter (3), Carcass weight (hot) (3), Leaf fat weight (3), Lean meat percentage (3), Meat color score (3), Muscle moisture percentage (3), NADPH-generating enzyme activity (3), PH for Longissmus dorsi (3), pH for Semimembranosus (3), Teat number (3), Backfat at tenth rib (2), Backfat thickness between 3rd and 4th rib (2), Backfat weight (2), Belly weight (2), Body weight (birth) (2), Cannon bone circumference (2), CIE-L* (2), Fat protein content (2), Japanese color scale (2), Loin muscle area (2), Loin muscle depth (2), Mean corpuscular volume (2), Shoulder subcutaneous fat thickness (2), Trimmed wholesale product / live weight (2), 3 hr-24 hr pH decline (1), 9 hr-24 hr pH decline (1), Abdominal fat weight (1), Age at puberty (1), Alkaline phosphatase activity (1), Average glycogen (1), backfat above muscle dorsi (1), Backfat at first rib (1), Backfat at rump (1), Backfat between 3rd and 4th last ribs (1), Body weight (end of test) (1), Body weight (slaughter) (1), Body weight (weaning) (1), Cervical vertebra length (1), Corpus luteum number (1), Dressing percentage (1), Drip loss (1), Ear size (1), Fat-cuts percentage (1), Fat area percentage in carcass (1), Feed conversion ratio (1), Feet weight (1), Ham weight (1), Head weight (1), Heart weight (1), Hematin pigmentation (1), hematocrit (1), Hemoglobin (1), Hemolytic complement activity (alternative pathway) (1), Left teat number (1), Liver weight (1), Loin and ham percentage in carcass (1), Loin fat percentage (1), Lymphocyte number (1), Mean corpuscular hemoglobin content (1), Meat color-a (1), Meat color-L (1), Percentage type I fibers (1), Percentage type IIb fibers (1), Red cell distribution width (1), Residual glycogen (1), Shear force at first peak (1), Shoulder weight (1), Skin percentage (1), Small intestine length (1), Smell intensity - fat (1), Subcutanous fat area (1), Time spent drinking (1), Time spent rooting (1), Time spent socializing (1) |
| 9 | MARC0029225 | rs81224033 | 64.93-65.33 | 1.4572 | *PLEKHA6,PPP1R15B,PIK3C2B, MDM4, LRRN2* | Shoulder weight (3), average daily gain (2), backfat at mid-back (2), Loin muscle area (2), Meat color-L (2), Off-Flavor Score (2), Palmitoleic acid content (2), Stearic acid content (2), androstenone laboratory (1), Average backfat thickness (1), backfat above muscle dorsi (1), Backfat at first rib (1), Backfat at tenth rib (1), CIE-a* (1), CIE-L* (1), CO2 partial pressure (1), Corpus luteum number (1), Diameter of type I muscle fibers (1), Drip loss (1), Ear erectness (1), Ear size (1), External fat on ham (1), External fat on loin (1), Feed intake (1), Ham fat weight (1), Haptoglobin concentration (1), Leaf fat weight (1), Melanoma susceptibility (1), Muscle moisture percentage (1), pH 40 minutes post mortem (ham) (1), Saturated fatty acid content (1), Shear force (1), Side fat thickness (1), Spleen weight (1) |
| 14 | MARC0002411 | rs81223780 | 83.8-84.2 | 1.6791 | *NRG3* | Fat androstenone level (4), Adipocyte diameter (3), Percentage type I fibers (3), Shear force at first peak (3), backfat at last rib (2), C3c concentration (2), Carcass length (2), Cervical vertebra length (2), Creatinine level (2), Ham percentage (2), Ham weight (2), Loin muscle area (2), Percentage type IIb fibers (2), Abdominal fat weight (1), Average backfat thickness (1), average daily gain (1), Average instron (star probe) force (1), backfat above muscle dorsi (1), Body weight (birth) (1), Body weight (slaughter) (1), Body weight (1), Calcium level (1), Carcass weight (cold) (1), Carcass weight (hot) (1), CIE-a* (1), CIE-b* (1), CIE-L* (1), Daily feed intake (1), Dressing percentage (1), External fat on ham (1), Fat-cuts percentage (1), Fat protein content (1), Feed intake (1), Haptoglobin concentration (1), Head weight (1), Heart weight (1), intestinal fat weight (1), Loin and neck meat weight (1), Meat color-a (1), Osteochondrosis score (1), pH 24 hr post-mortem (loin) (1), Potassium level (1), Salmonella count in liver and spleen (1), Salmonella count in liver (1), Salmonella count in spleen (1), Semimembranosus angle (1), Shear force (1), Shoulder meat weight (1), Shoulder subcutaneous fat thickness (1), Shoulder weight Boston (1), Total shear work (1), Trimmed wholesale product / carcass weight (1) |
| 14 | MARC0008412 | rs80838751 | 84.05-84.45 | 1.4959 | *NRG3* | Fat androstenone level (4), Adipocyte diameter (3), Percentage type I fibers (3), Shear force at first peak (3), backfat at last rib (2), C3c concentration (2), Carcass length (2), Creatinine level (2), Ham percentage (2), Ham weight (2), Loin muscle area (2), Percentage type IIb fibers (2), Abdominal fat weight (1), Average backfat thickness (1), average daily gain (1), Average instron (star probe) force (1), backfat above muscle dorsi (1), Body weight (birth) (1), Body weight (slaughter) (1), Body weight (1), Calcium level (1), Carcass weight (cold) (1), Carcass weight (hot) (1), Cervical vertebra length (1), CIE-a* (1), CIE-b* (1), CIE-L* (1), Daily feed intake (1), Dressing percentage (1), External fat on ham (1), Fat-cuts percentage (1), Fat protein content (1), Feed intake (1), Haptoglobin concentration (1), Head weight (1), Heart weight (1), intestinal fat weight (1), Loin and neck meat weight (1), Meat color-a (1), Osteochondrosis score (1), pH 24 hr post-mortem (loin) (1), Potassium level (1), Salmonella count in liver and spleen (1), Salmonella count in liver (1), Salmonella count in spleen (1), Semimembranosus angle (1), Shear force (1), Shoulder meat weight (1), Shoulder subcutaneous fat thickness (1), Shoulder weight Boston (1), Total shear work (1), Trimmed wholesale product / carcass weight (1) |
| 18 | ASGA0080224 | rs81470716 | 52.93-53.33 | 1.4355 | *--* | Actinobacillus pleuropneumoniae susceptibility (3), backfat at last rib (1), Corpus luteum number (1), Drip loss (1), Nonfunctional nipples (1), Teat number (1) |

^1^Chromosome.

**Table S5.** Summary of potential selection regions in Baoshan pigs (Ding’an and Tunchang/Baoshan group).

| **Chr^1^** | **SNP** | **ID** | **Position (Mb)** | **XP-EHH**  **score** | **Genes** | **QTL**  **(counts)** |
| --- | --- | --- | --- | --- | --- | --- |
| 10 | MARC0104534 | rs81280567 | 31.66-32.06 | -0.8453 | *FRMD3,*  *RASEF* | average daily gain (4), Dressing percentage (2), Percentage type I fibers (2), Average backfat thickness (1), backfat at mid-back (1), Carcass weight (hot) (1), Change in interferon-gamma level (1), CIE-a* (1), CIE-b* (1), Corpus luteum number (1), Creatinine level (1), External fat on loin (1), Gait score (hind) (1), Interleukin 10 level (1), Lumbar vertebra number (1), Marbling (1), Number of capillaries per muscle fiber (1), Number of capillaries per type IIa fiber (1), Osteochondrosis score (1), Percentage type IIa fibers (1), Semimembranosus angle (1), Shear force (1), Shoulder subcutaneous fat thickness (1), Teat number (1), Vertebra number (1) |

^1^Chromosome.

**Table S6.** Summary of potential selection regions in Hainan pigs (Ding’an and Tunchang/Saba group).

| **Chr^1^** | **SNP** | **ID** | **Position (Mb)** | **XP-EHH**  **score** | **Genes** | **QTL**  **(counts)** |
| --- | --- | --- | --- | --- | --- | --- |
| 1 | H3GA0004072 | rs80792171 | 268-268.4 | 0.8729 | *LRSAM1, FAM129B, STXBP1, CFAP157, PTRH1, TTC16, TOR2A, SH2D3C, CDK9, FPGS, ENG, AK1, ST6GALNAC6, ST6GALNAC4* | Drip loss (15), average daily gain (6), Teat number (6), backfat at last rib (3), Backfat weight (3), Carcass length (3), Fat-cuts percentage (3), White blood cell counts (3), Adipocyte diameter (2), Alkaline phosphatase activity (2), Average backfat thickness (2), Body weight (birth) (2), Body weight (weaning) (2), Fat percentage in carcass (2), Ham weight (2), Loin muscle area (2), Shoulder subcutaneous fat thickness (2), Spareribs weight (2), Vertebra number (2), Age at puberty (1), backfat above muscle dorsi (1), backfat at mid-back (1), Backfat at rump (1), Body weight (slaughter) (1), C3c concentration (1), Conductivity 24 hours postmortem (ham) (1), Diameter of type IIb muscle fibers (1), Dressing percentage (1), Ear erectness (1), External fat on ham (1), Fat area percentage in carcass (1), Fat weight (total) (1), Front feet conformation (1), Front leg conformation (1), Gait score (hind) (1), Gestation length (1), Jowl weight (1), Leaf fat weight (1), Lean cuts percentage (1), Lean meat percentage (1), Locomotion (1), Loin and neck meat weight (1), Loin fat percentage (1), Marbling (1), Meat color score (1), Muscle moisture percentage (1), Musculus biceps femoris and dissected ham ratio (1), Percentage type I fibers (1), pH 48 hr post-mortem (loin) (1), Physis score (1), Protein accretion rate (1), Shoulder external fat weight (1), Skin thickness (1), Thoracic vertebra number (1), Umbilical hernia (1) |
| 1 | ALGA0008961 | rs80943372 | 276.78-277.18 | 1.0013 | *--* | Drip loss (16), average daily gain (10), Teat number (5), Average backfat thickness (4), Backfat weight (4), Carcass length (3), Fat-cuts percentage (3), Ham weight (3), Loin muscle area (3), Shoulder subcutaneous fat thickness (3), Adipocyte diameter (2), Alkaline phosphatase activity (2), backfat at last rib (2), Body weight (weaning) (2), Fat percentage in carcass (2), Lean meat percentage (2), Loin weight (2), Spareribs weight (2), Vertebra number (2), White blood cell counts (2), Age at puberty (1), backfat above muscle dorsi (1), backfat at mid-back (1), Backfat at rump (1), body weight (10 weeks) (1), body weight (13 weeks) (1), body weight (17 weeks) (1), Body weight (birth) (1), Body weight (slaughter) (1), C3c concentration (1), Diameter of type IIb muscle fibers (1), Dressing percentage (1), Ear erectness (1), External fat on ham (1), Fat area percentage in carcass (1), Fat weight (total) (1), Front feet conformation (1), intestinal fat weight (1), Leaf fat weight (1), Lean cuts percentage (1), Locomotion (1), Loin and neck meat weight (1), Loin fat percentage (1), Marbling (1), Meat color score (1), Muscle moisture percentage (1), Percentage type I fibers (1), PH for Biceps femoris (1), PH for Longissmus dorsi (1), pH for Semispinalis Dorsi (1), Shoulder external fat weight (1), Skin thickness (1), Thoracic vertebra number (1), Trimmed wholesale product / carcass weight (1), Trimmed wholesale product / live weight (1), Umbilical hernia (1) |
| 1 | H3GA0004889 | rs80858349 | 299.48-299.88 | 0.8267 | *--* | Drip loss (16), Average backfat thickness (2), average daily gain (2), Backfat weight (2), body weight (13 weeks) (2), Ham weight (2), Loin weight (2), Trimmed wholesale product / live weight (2), Backfat at last lumbar (1), backfat at last rib (1), Backfat at tenth rib (1), body weight (10 weeks) (1), body weight (17 weeks) (1), Cannon bone circumference (1), Fat area percentage in carcass (1), Leaf fat weight (1), Lean meat percentage (1), Loin and ham percentage in carcass (1), Loin muscle area (1), PH for Biceps femoris (1), PH for Longissmus dorsi (1), pH for Semispinalis Dorsi (1), Trimmed wholesale product / carcass weight (1), Umbilical hernia (1) |
| 1 | H3GA0004945 | rs80819792 | 300.93-301.33 | 0.8705 | *--* | Drip loss (16), Average backfat thickness (2), average daily gain (2), Backfat weight (2), body weight (13 weeks) (2), Ham weight (2), Loin weight (2), Trimmed wholesale product / live weight (2), Backfat at last lumbar (1), backfat at last rib (1), Backfat at tenth rib (1), body weight (10 weeks) (1), body weight (17 weeks) (1), Cannon bone circumference (1), Fat area percentage in carcass (1), Leaf fat weight (1), Lean meat percentage (1), Loin and ham percentage in carcass (1), Loin muscle area (1), PH for Biceps femoris (1), PH for Longissmus dorsi (1), pH for Semispinalis Dorsi (1), Trimmed wholesale product / carcass weight (1), Umbilical hernia (1) |
| 2 | H3GA0006932 | rs81360002 | 82.66-83.06 | 1.1179 | *BTF3,ANKRA2,UTP15,ARHGEF28* | Actinobacillus pleuropneumoniae susceptibility (7), C3c concentration (4), Carcass weight (hot) (3), Cooking loss (3), Ham weight (3), Loin muscle area (3), Shear force (3), Shoulder subcutaneous fat thickness (3), Abdominal fat weight (2), average daily gain (2), backfat at last rib (2), backfat at mid-back (2), Drip loss (2), Estimated carcass lean content (2), Fat to meat ratio (2), Firmness (2), Off-Flavor Score (2), PH for Longissmus dorsi (2), tenderness score (2), Water holding capacity (2), androstenone laboratory (1), Average backfat thickness (1), Backfat at first rib (1), Backfat weight (1), Belly meat content (1), Body weight (16 days) (1), Body weight (end of test) (1), Carcass length (1), Carcass temperature (45 minutes post-mortem) (1), Carcass weight (cold) (1), Cholesterol level (1), CIE-b* (1), CIE-L* (1), Creatine kinase level (1), Cutlet weight (1), Diameter of muscle fibers (1), Diameter of type IIa muscle fibers (1), Diameter of type IIb muscle fibers (1), Fat-cuts percentage (1), Ham fat thickness (1), Intramuscular fat content (1), Loin muscle depth (1), Lumbar vertebra number (1), Meat color-L (1), Melanoma susceptibility (1), Muscle moisture percentage (1), Mycoplasma hyopneumoniae antibody titer (1), pH 24 hr post-mortem (loin) (1), pH 24 hr post mortem (ham) (1), pH for Semimembranosus (1), Platelet count (1), Red cell distribution width (1), Salmonella count in liver and spleen (1), Salmonella count in liver (1), Shear firmness (1), Shoulder external fat weight (1), Shoulder weight (1), Tetanus antibody titer (1) |
| 3 | MARC0062909 | rs81251364 | 48.93-49.33 | 0.9567 | *UXS1,C3H2orf40,NCK2* | average daily gain (6), Loin muscle area (3), Body weight (birth) (2), Carcass length (2), Carcass weight (hot) (2), CIE-b* (2), Fat androstenone level (2), Loin and neck meat weight (2), Nonfunctional nipples (2), pH 24 hr post-mortem (loin) (2), Base excess (1), Blood pH (1), body weight (17 weeks) (1), Body weight (34 weeks) (1), Body weight (end of test) (1), Carcass weight (cold) (1), CIE-a* (1), Cooking loss (1), Corpus luteum number (1), Cortisol level (1), Diameter of type IIb muscle fibers (1), Drip loss (1), Feed conversion ratio (1), Ham meat weight (1), Ham weight (1), Haptoglobin concentration (1), HDL cholesterol (1), Head weight (1), Lactate dehydrogenase level (1), Lactate level (1), Lean cuts percentage (1), Left teat number (1), Mean corpuscular volume (1), Meat color chroma (1), Number of capillaries per type I fiber (1), Number of muscle fibers per unit area (1), Off-Flavor Score (1), Osteochondrosis score (1), pH 45 minutes post mortem (1), Shear force (1), Shoulder meat weight (1), Side fat thickness (1), Teat number (1), Total shear work (1), White blood cell counts (1) |
| 3 | MARC0063543 | rs81251441 | 48.94-49.34 | 1.0670 | *UXS1,C3H2orf40,NCK2* | average daily gain (6), Loin muscle area (3), Body weight (birth) (2), Carcass length (2), Carcass weight (hot) (2), CIE-b* (2), Fat androstenone level (2), Loin and neck meat weight (2), Nonfunctional nipples (2), pH 24 hr post-mortem (loin) (2), Base excess (1), Blood pH (1), body weight (17 weeks) (1), Body weight (34 weeks) (1), Body weight (end of test) (1), Carcass weight (cold) (1), CIE-a* (1), Cooking loss (1), Corpus luteum number (1), Cortisol level (1), Diameter of type IIb muscle fibers (1), Drip loss (1), Feed conversion ratio (1), Ham meat weight (1), Ham weight (1), Haptoglobin concentration (1), HDL cholesterol (1), Head weight (1), Lactate dehydrogenase level (1), Lactate level (1), Lean cuts percentage (1), Left teat number (1), Mean corpuscular volume (1), Meat color chroma (1), Number of capillaries per type I fiber (1), Number of muscle fibers per unit area (1), Off-Flavor Score (1), Osteochondrosis score (1), pH 45 minutes post mortem (1), Shear force (1), Shoulder meat weight (1), Side fat thickness (1), Teat number (1), Total shear work (1), White blood cell counts (1) |
| 12 | ASGA0054041 | rs81433573 | 32.55-32.95 | 0.8425 | *ANKFN1,NOG* | Muscle moisture percentage (4), Teat number (4), Actinobacillus pleuropneumoniae susceptibility (3), Ear erectness (3), Body weight (birth) (2), Diameter of type I muscle fibers (2), pH 24 hr post-mortem (loin) (2), Average backfat thickness (1), backfat at last rib (1), backfat at mid-back (1), Belly weight (1), body weight (10 weeks) (1), Body weight (20 weeks) (1), Carcass weight (hot) (1), CIE-a* (1), Conductivity 24 hours postmortem (loin) (1), Creatine kinase level (1), Drip loss (1), Fat percentage in carcass (1), Ham fat thickness (1), Interleukin 10 level (1), Leaf fat weight (1), Left teat number (1), Loin muscle area (1), Loin muscle depth (1), Loin weight (1), Meat color-L (1), Muscle fat content (1), Number of muscle fibers per unit area (1), pH 45 minutes post mortem (1), Phosphate level (1), Red cell distribution width (1), Right teat number (1), Thoracic vertebra number (1), Toll-like receptor 2 level (1), Toll-like receptor 9 level (1), Total muscle fiber number (1), Triglyceride level (1), Vertebra number (1) |
| 13 | H3GA0038005 | rs80782255 | 210.64-211.04 | 0.8679 | *--* | Body weight (5 weeks) (1), Cortisol level (1), Melanoma susceptibility (1), Percentage type IIa fibers (1) |
| 14 | MARC0002411 | rs81223780 | 83.8-84.2 | 0.9912 | *NRG3* | Fat androstenone level (4), Adipocyte diameter (3), Percentage type I fibers (3), Shear force at first peak (3), backfat at last rib (2), C3c concentration (2), Carcass length (2), Cervical vertebra length (2), Creatinine level (2), Ham percentage (2), Ham weight (2), Loin muscle area (2), Percentage type IIb fibers (2), Abdominal fat weight (1), Average backfat thickness (1), average daily gain (1), Average instron (star probe) force (1), backfat above muscle dorsi (1), Body weight (birth) (1), Body weight (slaughter) (1), Body weight (1), Calcium level (1), Carcass weight (cold) (1), Carcass weight (hot) (1), CIE-a* (1), CIE-b* (1), CIE-L* (1), Daily feed intake (1), Dressing percentage (1), External fat on ham (1), Fat-cuts percentage (1), Fat protein content (1), Feed intake (1), Haptoglobin concentration (1), Head weight (1), Heart weight (1), intestinal fat weight (1), Loin and neck meat weight (1), Meat color-a (1), Osteochondrosis score (1), pH 24 hr post-mortem (loin) (1), Potassium level (1), Salmonella count in liver and spleen (1), Salmonella count in liver (1), Salmonella count in spleen (1), Semimembranosus angle (1), Shear force (1), Shoulder meat weight (1), Shoulder subcutaneous fat thickness (1), Shoulder weight Boston (1), Total shear work (1), Trimmed wholesale product / carcass weight (1) |
| 14 | MARC0008412 | rs80838751 | 84.05-84.45 | 0.9015 | *NRG3* | Fat androstenone level (4), Adipocyte diameter (3), Percentage type I fibers (3), Shear force at first peak (3), backfat at last rib (2), C3c concentration (2), Carcass length (2), Creatinine level (2), Ham percentage (2), Ham weight (2), Loin muscle area (2), Percentage type IIb fibers (2), Abdominal fat weight (1), Average backfat thickness (1), average daily gain (1), Average instron (star probe) force (1), backfat above muscle dorsi (1), Body weight (birth) (1), Body weight (slaughter) (1), Body weight (1), Calcium level (1), Carcass weight (cold) (1), Carcass weight (hot) (1), Cervical vertebra length (1), CIE-a* (1), CIE-b* (1), CIE-L* (1), Daily feed intake (1), Dressing percentage (1), External fat on ham (1), Fat-cuts percentage (1), Fat protein content (1), Feed intake (1), Haptoglobin concentration (1), Head weight (1), Heart weight (1), intestinal fat weight (1), Loin and neck meat weight (1), Meat color-a (1), Osteochondrosis score (1), pH 24 hr post-mortem (loin) (1), Potassium level (1), Salmonella count in liver and spleen (1), Salmonella count in liver (1), Salmonella count in spleen (1), Semimembranosus angle (1), Shear force (1), Shoulder meat weight (1), Shoulder subcutaneous fat thickness (1), Shoulder weight Boston (1), Total shear work (1), Trimmed wholesale product / carcass weight (1) |

^1^Chromosome.

**Table S7.** Summary of potential selection regions in Saba pigs (Ding’an and Tunchang/Saba group).

| **Chr^1^** | **SNP** | **ID** | **Position (Mb)** | **XP-EHH**  **score** | **Genes** | **QTL**  **(counts)** |
| --- | --- | --- | --- | --- | --- | --- |
| 10 | MARC0104534 | rs81280567 | 31.66-32.06 | -0.9665 | *FRMD3,*  *RASEF* | average daily gain (4), Dressing percentage (2), Percentage type I fibers (2), Average backfat thickness (1), backfat at mid-back (1), Carcass weight (hot) (1), Change in interferon-gamma level (1), CIE-a* (1), CIE-b* (1), Corpus luteum number (1), Creatinine level (1), External fat on loin (1), Gait score (hind) (1), Interleukin 10 level (1), Lumbar vertebra number (1), Marbling (1), Number of capillaries per muscle fiber (1), Number of capillaries per type IIa fiber (1), Osteochondrosis score (1), Percentage type IIa fibers (1), Semimembranosus angle (1), Shear force (1), Shoulder subcutaneous fat thickness (1), Teat number (1), Vertebra number (1) |

^1^Chromosome.
